# Secondary Contact, Introgressive Hybridization, and Genome Stabilization in Sticklebacks

**DOI:** 10.1093/molbev/msae031

**Published:** 2024-02-15

**Authors:** Xueyun Feng, Juha Merilä, Ari Löytynoja

**Affiliations:** Organismal and Evolutionary Biology Research Programme, Faculty of Biological and Environmental Sciences, University of Helsinki, Helsinki 00014, Finland; Institute of Biotechnology, University of Helsinki, Helsinki 00014, Finland; Organismal and Evolutionary Biology Research Programme, Faculty of Biological and Environmental Sciences, University of Helsinki, Helsinki 00014, Finland; Area of Ecology and Biodiversity, The School of Biological Sciences, Kadoorie Biological Sciences Building, The University of Hong Kong, Hong Kong, Hong Kong SAR; Institute of Biotechnology, University of Helsinki, Helsinki 00014, Finland

**Keywords:** adaptation, admixture, gene flow, hybridization, recombination rate, secondary contact

## Abstract

Advances in genomic studies have revealed that hybridization in nature is pervasive and raised questions about the dynamics of different genetic and evolutionary factors following the initial hybridization event. While recent research has proposed that the genomic outcomes of hybridization might be predictable to some extent, many uncertainties remain. With comprehensive whole-genome sequence data, we investigated the genetic introgression between 2 divergent lineages of 9-spined sticklebacks (*Pungitius pungitius*) in the Baltic Sea. We found that the intensity and direction of selection on the introgressed variation has varied across different genomic elements: while functionally important regions displayed reduced rates of introgression, promoter regions showed enrichment. Despite the general trend of negative selection, we identified specific genomic regions that were enriched for introgressed variants, and within these regions, we detected footprints of selection, indicating adaptive introgression. Geographically, we found the selection against the functional changes to be strongest in the vicinity of the secondary contact zone and weaken as a function of distance from the initial contact. Altogether, the results suggest that the stabilization of introgressed variation in the genomes is a complex, multistage process involving both negative and positive selection. In spite of the predominance of negative selection against introgressed variants, we also found evidence for adaptive introgression variants likely associated with adaptation to Baltic Sea environmental conditions.

## Introduction

Introgression is a process that transfers genetic variation from 1 species or divergent lineage to another. Whole-genome analyses have shown introgression to be an important and pervasive evolutionary force (e.g. Mallet 2005; Lamichhaney et al. 2018; Oziolor et al. 2019) that has shaped the genome of many organisms (e.g. Dowling and Secor 1997; Harrison and Larson 2014; Suarez-Gonzalez et al. 2018), including humans (Huerta-Sánchez et al. 2014; Sankararaman et al. 2014, 2016). There is also well-documented evidence of introgression fueling adaptation in several species (Hedrick 2013; Racimo et al. 2015; Marques et al. 2019; Wang et al. 2023). However, amidst this process of introgression of adaptive and neutral variants, there exists a genome-wide selection against hybrids (e.g. Arnegard et al. 2014; Christie and Strauss 2018) and regions derived from hybridization throughout the entire genome (Harrison and Larson 2014; Sankararaman et al. 2014; Juric et al. 2016; Schumer et al. 2018; Calfee et al. 2021). The seemingly conflicting observations of widespread hybridization in nature and the prevalence of selection against foreign ancestry can be explained by the various factors contributing to the reduced fitness of hybrids: In addition to ecological selection against hybrids, the hybridizing parental populations may carry harmful variants (hybridization load), or the genes of the 2 parental lineages may have negative interactions (hybrid incompatibilities) (Moran et al. 2021).

Genomic studies of contemporary hybrids have shown that the proportion of foreign ancestry is highly variable among species (Martin et al. 2015, 2019; Malinsky et al. 2018) and populations (Skoglund et al. 2015; Kuhlwilm et al. 2016), and the introgressed ancestry is unevenly distributed across the genome (Sankararaman et al. 2014, 2016; Vattathil and Akey 2015; Zhang et al. 2016). However, the mechanisms underlying this heterogeneous distribution are not well understood. Generally, introgressed alleles are regarded to have a negative fitness effect when introduced into new genomic backgrounds (Amorim et al. 2017; Martin and Jiggins 2017; Bay et al. 2019), and according to the Dobzhansky–Muller model of hybrid incompatibility (Bomblies et al. 2007; Masly and Presgraves 2007; Lee et al. 2008), long-term negative selection on incompatible loci may create “deserts” of introgression in the genome (Sankararaman et al. 2014, 2016). However, genetic architecture and constraints also play a role, and genomic regions characterized by higher gene density and/or low recombination rate are expected to show a lower rate of introgression than other regions (Martin and Jiggins 2017). This prediction is well supported by empirical studies in humans (Sankararaman et al. 2014, 2016), fish (Schumer et al. 2018), and butterflies (Edelman et al. 2019; Martin et al. 2019), and the intensity of selection against introgression in these studies appears to be positively correlated with the density of functional elements. Nevertheless, the intricate interactions between the different forces against foreign ancestry and the predictability of the ultimate outcomes of hybridization are yet to be fully comprehended.

Introgression and admixture always happen in an evolutionary context, and the hybridizing lineages can differ in terms of their demographic histories and population sizes, levels of genetic drift, and strength of selection against deleterious variants prior to the hybridization event (Schumer et al. 2018; Moran et al. 2021; Liu et al. 2022). Gene flow from a population with a smaller effective population size (*N*_e_) and reduced purifying selection efficiency may increase the genetic load in the hybrid population through the introduction of weakly deleterious alleles (Harris and Nielsen 2016; Juric et al. 2016). Typically, gene flow from a population with a larger *N*_e_ is thought to ease the genetic load (Edelman and Mallet 2021), but in extreme cases, it may import unbearable amounts of recessive lethal variation and condemn a tiny population into extinction (Kyriazis et al. 2021). Consequently, the selection on introgressed variants, both adaptive and maladaptive, plays a pivotal role in shaping the genome-wide patterns of foreign ancestry (Kim et al. 2018). The simultaneous operation of multiple demographic and selective processes may lead to interwoven effects, emphasizing the need for a systematic investigation of the historical demographic events and the distinct evolutionary forces that shape the genomic landscape of introgression. A thorough understanding of both the history and the genomic mechanisms is vital for comprehending the evolutionary consequences of hybridization.

The 9-spined stickleback (*Pungitius pungitius*) is a small euryhaline teleost fish that inhabits circumpolar regions of the northern hemisphere. The evolutionary history of 9-spined sticklebacks has been extensively studied (Aldenhoven et al. 2010; Shikano, Ramadevi, et al. 2010; Teacher et al. 2011; Bruneaux et al. 2013; Guo et al. 2019; Natri et al. 2019; Feng et al. 2022, 2023), and in Europe, 2 distinct evolutionary lineages have been identified: the Western European lineage (WL) and the Eastern European lineage (EL). Despite the lineages exhibiting distinct sex determination systems (Natri et al. 2019) and highly differentiated mitochondrial haplotypes (Aldenhoven et al. 2010; Shikano, Shimada, et al. 2010; Guo et al. 2019), they are known to interbreed (Natri et al. 2019) and populations in the Baltic Sea area display a gradient of mixed ancestry (Feng et al. 2022). The identity of the participating populations and the exact timing of the Baltic Sea admixture event(s) are unknown. Based on purely geographical information (Ukkonen et al. 2014), the EL appears to have colonized the Baltic Sea relatively late (in the Ancylus Lake stage, 10,700 to 10,200 BP; Björck 2008; Feng et al. 2022) and it seems likely that the large water body had (WL origin) sticklebacks trapped for the duration of the ice age or at least the species had colonized the area during the Yoldia Sea stage (11,600 to 10,700 BP). If the area was inhabited by WL-origin sticklebacks, the 2 lineages met the first time when the EL colonized the Baltic Sea; the second window for admixture started after the reopening of the Danish Straits during the Littorina Sea stage (at the latest 8,500 to 6,000 BP). If the pre-Ancylus waters were void of sticklebacks, the area was first of EL-origin and the 2 lineages have admixed during or after the Littorina Sea stage and the formation of the current Baltic Sea.

The Baltic Sea is a relatively shallow brackish water inland sea with steep gradients of salinity and other abiotic factors (Szaniawska 2018). Due to its young age and recent colonization by various species, it provides an appealing system to study adaptation to variable conditions (Reusch et al. 2018). The genetic resources, the broad-scale sampling, and the detailed demographic history available for the Baltic Sea 9-spined sticklebacks (Varadharajan et al. 2019; Kivikoski et al. 2021; Feng et al. 2022) provide a good starting point for separating the complex signals of various evolutionary factors. The known genetic and phenotypic variation in the area (Herczeg et al. 2009, 2010; Shikano, Ramadevi, et al. 2010; Teacher et al. 2011; Guo et al. 2019; Natri et al. 2019; Feng et al. 2022, 2023) makes the species an intriguing model for investigating admixture, and the presence of populations with large *N*_e_ reduces the impact of drift (Pamilo and Nei 1988; Palumbi 1994; Tigano and Friesen 2016; Jokinen et al. 2019) and the hybridization load and thus permits excluding specific mechanisms from the process. Ideally, the Baltic Sea sticklebacks should allow studying the consequences of admixture and the stabilization of the postadmixture genome and help understand the interplay of different forces and mechanisms that lead genomes to resist ongoing introgression in hybrid zones.

The objective of this study was to investigate the mechanisms influencing the introgression landscape between 2 evolutionary lineages of 9-spined sticklebacks (*P. pungitius*) after their secondary contact in northern Europe. To this end, we employed whole-genome sequence data from 284 individuals belonging to 13 populations across the Baltic Sea hybrid zone, subsampled from Feng et al. (2023). Our analysis aimed to estimate the levels of introgression across various populations and different genomic regions, thereby characterizing the genomic landscape of introgression across the hybrid zone. We had three main objectives: (i) to comprehend the leading evolutionary forces that have shaped the genomic landscape of introgression, (ii) to identify genomic regions where positive natural selection has likely favored introgressed genetic elements, and (iii) to elucidate how natural selection has influenced the genome-wide patterns of introgression, particularly regarding the transfer and elimination of adaptive and deleterious variants between the 2 lineages. Our findings suggest that a diverse array of evolutionary forces has contributed to shaping the genomic landscape of introgression, the fast removal of highly deleterious variations, and the long-term selection against weak deleterious variations being the predominant driving forces. The different sources of selection have interacted with the variable recombination landscape and genome structure, thereby adding complexity to the predictability of postadmixture genome evolution in the hybrids.

## Results

### Data Description

**Fig. 1.**
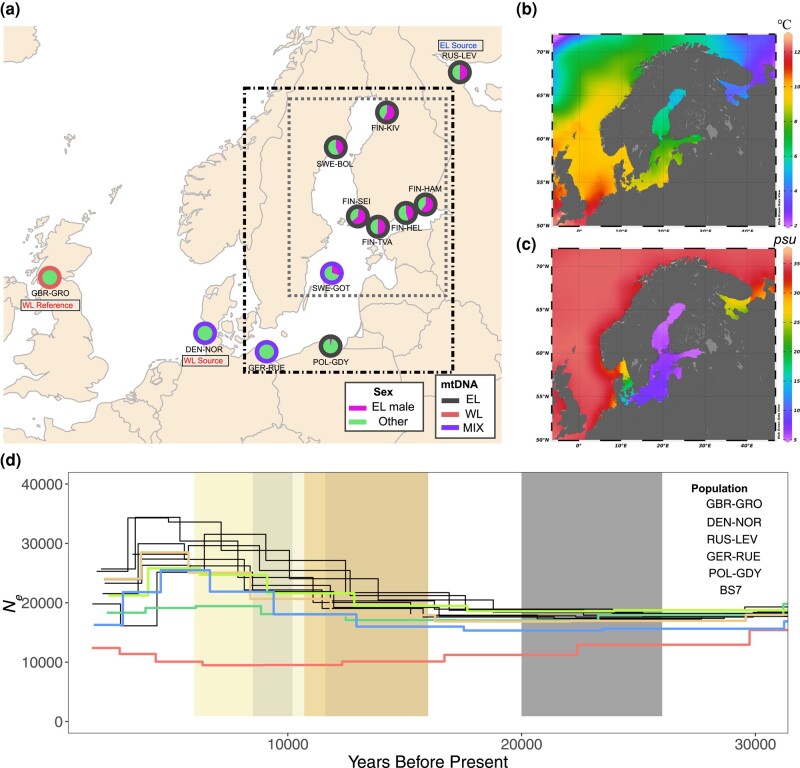
Study populations and localities. (a) Geographic origins of populations involved in this study, modified from Feng et al. (2022). The pie charts show the proportions of Eastern Lineage (EL) males; “Other” can be either WL male or WL/EL female. The outline colors indicate the mtDNA lineage assignment of the population. The dot-dashed frame marks the admixed populations, and the gray dotted frame indicates the BS7 set (see Materials and Methods). The source and reference populations used in the *f4-ratio* test and *fd* statistics are indicated. (b) Map of the sea surface temperature and (c) salinity of the Baltic Sea and its surroundings, adapted from Schlitzer and Mieruch-Schnülle (2024). (d) Demographic history of parental, reference, and admixed populations from 30,000 yr ago (kya) to the present, adapted from Feng et al. (2023). The blue, green, and red colors indicate the EL parental population RUS-LEV, WL parental population DEN-NOR, and WL reference population GBR-GRO, respectively; the orange and green colors indicate GER-RUE and POL-GDY, the 2 southern Baltic Sea populations, respectively. The 7 populations from the central and northern Baltic Sea are depicted in black. The yellow shadings indicate different stages of the Baltic Sea: the Baltic Ice Lake (16,000 to 11,600 BC), the Yoldia Sea (11,600 to 10,700 BC), the Ancylus Lake (10,700 to 10,200 BC), the fresh-to-brackish water transition stage (10,200 to 8,500), and the Littorina Sea (8,500 to 6,000 BC). The gray shading indicates the last glacial maximum (26,000 to 20,000 BC).

We retained 284 individuals from 13 populations of the total data of 888 individuals and 45 populations studied earlier by Feng et al. (2022 & 2023) (Fig. 1a and supplementary table S1, Supplementary Material online). The admixed populations were required to be from marine localities with variation in environmental conditions (Fig. 1b and c) and have large *N*_e_ (Fig. 1d; Feng et al. 2023) to minimize the impact of genetic drift. After quality control, 4,294,816 biallelic single-nucleotide polymorphisms (SNPs) over the 20 autosomal linkage groups (LGs) remained for the following analyses.

### Quantification of Introgression across the Genome

**Fig. 2.**
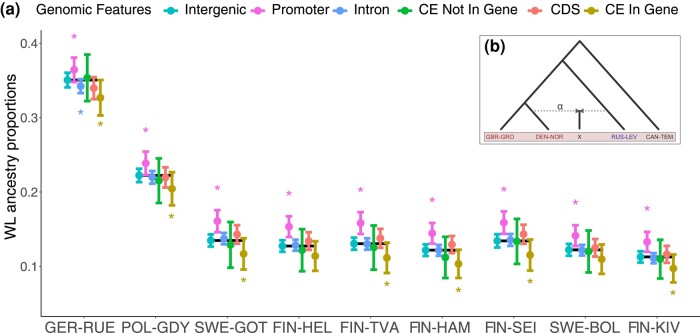
Western lineage ancestry across 6 different genomic features in the admixed Baltic Sea populations. (a) The dots and lines show the proportion of WL ancestry and its standard error (estimated with jackknifing across the genome) for each genomic feature, and the asterisks above and below indicate that the estimate is significantly higher or lower than within the intergenic region. The black lines indicate the estimates for intergenic regions, assumed to represent the background level of WL ancestry. The populations are ordered by increasing geographic distance from the Danish Straits. CE, constrained elements; CDS, coding sequences. (b) The tree depicts the setup of the *f4-ratio* test for assessing the WL ancestry following Petr et al. (2019). The test population X is assumed to be a mixture between populations DEN-NOR and RUS-LEV, α indicating the amount of WL ancestry.

Following Petr et al. (2019), we applied the *f_4_-ratio* test (Reich et al. 2009) to quantify the composition of WL ancestries in the Baltic Sea populations. In the intergenic regions, considered representing the background level, the southern Baltic Sea populations (GER-RUE and POL-GDY) contain 35% and 22.2% of WL ancestry (Fig. 2a), respectively, whereas the more northern populations from Gotland (SWE-GOT), Gulf of Finland (FIN-HEL and others), and the Bothnian Bay (SWE-BOL, FIN-KIV) contain 13.5% to 11.3% of WL ancestry, consistent with the whole-genome estimates of Feng et al. (2022). Overall, the ancestry proportions show a gradient across the Baltic Sea with the foreign ancestry decreasing with increasing distance from the Danish straits (Fig. 2a, supplementary table S2, Supplementary Material online).

To examine the potential impacts of selection on the minor parental ancestries, we binned the genome into functional categories and computed the WL ancestries across them using the *f_4_-ratio* test. We considered 6 categories: intergenic, coding DNA (CDS), constrained elements located inside or outside of genes, introns, and promoters. According to these estimates, all Baltic Sea populations contain significantly elevated amounts of WL ancestry in promoter regions (*P* < 0.001 to 0.044, estimated via resampling, see Materials and Methods; supplementary table S2, Supplementary Material online). Additionally, except for FIN-HEL and SWE-BOL (*P* = 0.090 to 0.098), all populations display significantly lower amounts of WL ancestry in constrained elements located within genes (*P* = 0.024 to 0.055). While not statistically significant for all mid to northern Baltic Sea populations, there was a slight increase in WL ancestry in CDS compared with intergenic regions.

### Footprints of Selection on WL Introgression in Baltic Sea Populations

**Fig. 3.**
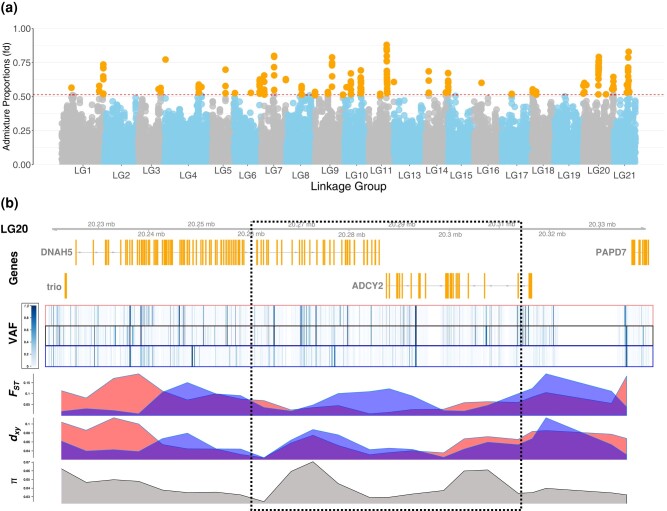
AI in the northern Baltic Sea populations. (a) The Manhattan plot shows the estimated admixture proportions (fd) for 100 kb windows across the genome, orange dots indicating genomic windows significantly enriched for WL ancestry. The dotted red line at 0.5137 indicates the *P* < 0.05 significance level. (b) A candidate region for AI at LG20:20260000-20315000 (dotted box). The panels show the gene annotations (coding sequences in orange), per site VAF (heatmap) for the 3 sets of population (WL source, BS7, EL source), and *F*_ST_, *d*_xy_, and *π* (10 kb windows) in the northern Baltic Sea populations (BS7). The red and blue colors indicate *F*_ST_ and *d*_xy_ measured against DEN-NOR (WL source) and RUS-LEV (EL source), respectively.

The 7 populations from the northern Baltic Sea showed similar levels of genetic introgression and were studied more closely to understand the factors shaping the genomic landscape of introgression as well as the potential adaptive nature of the introgressed variation. To more precisely identify the regions enriched with introgressed WL variants, we combined the populations, referred to as BS7, and computed the *fd* summary statistic (Martin et al. 2015) for 100 kb windows with 20 kb steps across the genome. Based on the false discovery rate (FDR) corrected *P*-value cutoff at 0.05, we obtained 181 putative introgression-enriched regions; by merging the overlapping regions, these collapsed into 45 regions with lengths varying from 100 to 560 kb (Fig. 3a, supplementary table S3, Supplementary Material online).

By definition, introgression introduces novel variation to a population and the footprints of selection within introgressed genomic regions differ from those expected under models without introgression (Setter et al. 2020). More precisely, methods based on polymorphism patterns may fail to detect the signals of selection, and approaches based on the overrepresentation of introgressed alleles in a specific population relative to other populations are considered more robust (Racimo et al. 2015). Following this, adaptive introgression (AI) can be distinguished from neutral admixture using the number of sites uniquely shared between the donor and recipient population (*U* test) as well as the allele frequencies on those sites (*Q95* test; Racimo et al. 2016). We applied the *U* and *Q95* tests to search for footprints of selection amongst the introgressed variants and obtained 44 regions which collapsed into 11 candidate regions (supplementary table S4, Supplementary Material online). Five of these candidate regions overlapped with the regions identified with the *fd* analysis and were chosen as candidates for AI. Integrating information from *F*_ST_, *d*_xy_, variant allele frequencies (VAF), and genetic diversity (*π*; Martin et al. 2015, Setter et al. 2020), we identified 4 candidates for adaptively introgressed genes (viz. *ZP4*, *PLEKHG3*, *DNAH5*, *ADCY2*; supplementary table S4 and fig. S2, Supplementary Material online). In particular, we searched for signals within the introgression-enriched region that exhibit lower *F*_ST_ and *d*_xy_ to the WL reference than the neighboring regions (Martin et al. 2015). Additionally, we expected the genetic diversity to display the characteristic “volcano” pattern (Setter et al. 2020) and the allele frequencies to be similar to those in the WL. The region in LG20 shows all the hallmarks of a selective sweep (Fig. 3b): lowered *F*_ST_ to the WL reference and increased *F*_ST_ to the EL reference, no significant increase in *d*_xy_, and a volcano-shaped pattern of *π* created by recombination between the alternative haplotypes. Although the number of genes found within the candidate regions is small, this does not exclude the possibility that a greater number of genes would be under adaptive selection, e.g. through introgressed promoter regions or other regulatory elements and structural variations.

### Interaction between Introgression, Differentiation, and Recombination Rate

Possible mechanisms causing heterogeneity in admixture proportions and differentiation across the recipient populations' genomes include incompatibility of genetic variants introgressed from a diverged lineage (Schumer et al. 2018), selection against introduced deleterious variation (Juric et al. 2016; Kovach et al. 2016), and adaptive evolution in different environments (Chunco 2014; Smukowski Heil et al. 2019). As selection removes negative variants, it also removes linked neutral variation, giving the variation in recombination rate a role in shaping the distribution and patterns of introgression across the genome (e.g. Schumer et al. 2018). In our data, WL ancestry proportion and recombination showed positive correlation only in the German coastal population (supplementary fig. S3, Supplementary Material online) and even there the correlation was weak (*r*_s_ = 0.064, *P* < 0.001). Interestingly, no correlation was found in the Polish population (*r*_s_ = −0.029, *P* = 0.082) and in Gotland (*r*_s_ = −0.023, *P* = 0.174) but a weak negative correlation was seen in the northern Baltic Sea populations (*r*_s_ = −0.090 to −0.039, *P ≤* 0.018). In the combined BS7 set, the correlation was slightly negative (*r*_s_ = −0.046, *P* = 0.006; Table 1).

**Table 1:** Spearman rank correlations between admixture proportion (*fd*) and recombination rate in different populations

Population	*r*_s_	*P*
GER-RUE	0.064	0.0001
POL-GDY	−0.029	0.0822
SWE-GOT	−0.023	0.1739
FIN-HEL	−0.044	0.0103
FIN-HAM	−0.090	2.3e-7
FIN-TVA	−0.059	0.0006
FIN-SEI	−0.041	0.0176
SWE-BOL	−0.051	0.0033
FIN-KIV	−0.039	0.0233
BS7	−0.046	0.0064

BS7, combined 7 northern Baltic Sea populations.

Due to decreased *N*_e_ (and increased drift) caused by background selection, genomic regions experiencing lower levels of recombination are expected to be more differentiated than those experiencing more recombination (Nachman and Payseur 2012). In our data, the genetic diversity and the genetic distance measurements *F*_ST_ and *d*_xy_, regardless of which parental population they were compared with, were always positively correlated with recombination rate (*r*_s_ = 0.179 to 0.615, *P ≤* 0.001; supplementary table S5, Supplementary Material online). As the density of coding sequences across the genome is weakly and positively correlated with the recombination rate (*r*_s_ = 0.098, *P* < 0.001), it is not surprising to see the admixture proportion also weakly correlating with the density of coding sequences (*r*_s_ = −0.047, *P* = 0.006) and density of constrained elements (*r*_s_ = −0.074, *P* < 0.001).

### Introgressive Genetic Load across Populations

**Fig. 4.**
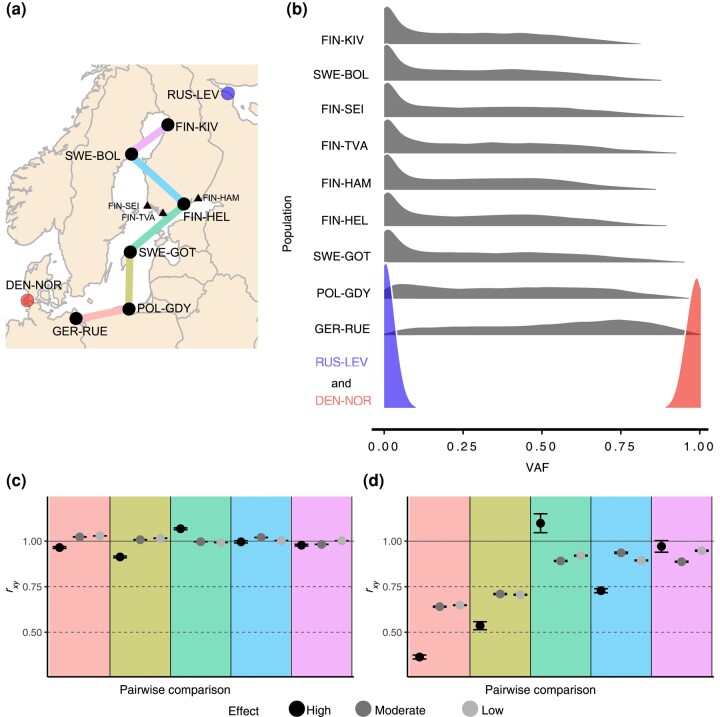
Selection on potentially deleterious introgressed variation. (a) Populations for the pairwise *r*_xy_ comparisons with colors matching those in panels c and d. The 3 populations from the Gulf of Finland indicated with black triangles were not included in the pairwise comparisons. The populations representing the WL and EL parental lineages are indicated with red and blue dots, respectively. (b) Density plots of the WL-origin VAF in the admixed Baltic Sea populations and the 2 parental populations. (c) and (d) The *r*_xy_ statistics for all (c) and WL-origin (d) coding variants of different impact for the 5 pairwise comparisons (supplementary table S6, Supplementary Material online). *r*_xy_ = 1 if the allele frequency changes do not deviate from the background; *r*_xy_ < 1 indicates the relative deficit of the corresponding variants in the northern population compared with the southern population. *r*_xy_ confidence intervals are based on jackknife estimates across the 20 linkage groups.

Given the dramatic differences in WL ancestry across the localities and the different genomic features, we set out to investigate the steps, progression, and stabilization of the WL ancestry within the genome since the secondary contact. Specifically, we investigated how the efficacy of selection on introgressed variants changes across the Baltic Sea (Fig. 4a). First, we identified WL-origin variants with opposing allele frequencies (Fig. 4b); these alleles were found in high frequencies in the south, but their frequency dramatically decreased in the central Baltic Sea and further declined toward the north. Second, we estimated the *r*_xy_ statistics for all coding variants and the WL-origin coding variants for population pairs with increasing distance from the Danish Straits. The results show that, while no apparent differences are seen at the genome-wide level (Fig. 4c), the selection has very efficiently purged the introgressed genetic variation in the south compared with the mid and northern Baltic Sea (Fig. 4d), especially the variants inferred to have a significant effect and causing early stop codons (high impact). Interestingly, we found that the strength of purging of WL-origin variation is not much different between synonymous (low impact) and nonsynonymous (moderate impact) variants. The efficiency of the purging of WL-origin variation does not appear to depend on the recombination rate (*r*_s_ = −0.176, *P* = 0.220; supplementary fig. S4, Supplementary Material online).

## Discussion

In hybridization, 2 locally adapted genomes get mixed and the intragenomic interactions get broken, opening a window for exceptionally dynamic evolution followed by a phase of subsequent stabilization. Large-scale DNA sequencing has revealed the prevalence of genetic introgression in the wild, but the events after the hybridization and the roles and the interplay of the different evolutionary factors are more difficult to study and still poorly understood. While some basic principles of hybridization have been emerging (Moran et al. 2021) and it is well documented that the foreign ancestry is selected against within the functionally most important genome regions, little is known about the relative importance of the different evolutionary forces and their interactions and, ultimately, how predictable the outcomes of hybridization are.

We investigated the genome-wide patterns of genetic introgression between 2 divergent lineages of 9-spined sticklebacks across the Baltic Sea and found the minor parental ancestry being generally selected against, with only a few regions showing signals of selection favoring the foreign ancestry. We found little correlation between the admixture proportion and the recombination rate, indicating a limited role for recombination in shaping the genomic landscape of introgression in this model system. Although we cannot fully separate the signals created by the temporal factors of a potentially multistaged admixture history from those created by the selection, the results highlight the complex forces acting in hybrid populations and demonstrate the potential of the Baltic Sea sticklebacks as a model system to study the evolutionary processes after secondary contacts in action.

### Genomic Variation of Minor Parental Ancestry

In hybrid populations, genome-wide patterns of ancestry are predicted to be extremely variable right after the hybridization event and then gradually stabilize over time. In our study, the WL ancestry decreased from 35% in the population closest to the entry to the Atlantic to 22.2% in a population 330 km to the east and then leveled at 13.5% to 11.3% in mid and northern Baltic Sea populations. In principle, this could be explained by the ∼12% WL ancestry in the mid and northern parts of the Baltic Sea representing the ancestral, first-stage admixture event and the higher WL ancestry levels in the south being explained by more recent and still ongoing WL migration. However, consistent with a number of previous studies showing that hybridization is selected against (Arnegard et al. 2014; Sankararaman et al. 2014; Harris and Nielsen 2016; Juric et al. 2016; Christie and Strauss 2018; Schumer et al. 2018; Calfee et al. 2021), we found that the selection has specifically removed the functionally significant WL ancestry in the southern Baltic Sea, both in the most conserved parts of coding sequences (Fig. 2a) and among functional coding sequence changes (Fig. 4d). While the genome-wide ancestry proportions in the mid and northern parts of the Baltic Sea are highly similar, there is a consistent pattern of WL ancestry being enriched in the promoter regions and being depleted in the constrained elements within coding sequences. Consistent with the latter, the *r*_xy_ values for the amino-acid-changing variants of WL origin are below 1 in all pairwise comparisons, indicating selection against the functionally significant foreign variation. Similar patterns of rapid removal of largely deleterious introgressed variation have been found in studies of hominins, swordtail fishes, and *Drosophila* (Harris and Nielsen 2016; Schumer et al. 2018; Matute et al. 2020; Veller et al. 2023), and such a pattern is considered to be widespread.

### Diverse Evolutionary Forces Shaped the Landscape of Introgression

Recombination rate variation is known to play a key role in shaping the genomic landscape of introgression (e.g. Kim et al. 2018; Martin et al. 2019; Veller et al. 2023). Generally, the footprints of selection are more prominent in regions with low recombination rates where the minor parental haplotypes are longer and thus more likely to contain harmful alleles (Wu, 2001; Nachman and Payseur 2012). As a result, a positive correlation between the admixture proportion and recombination rate is expected if the selection against genomic incompatibilities is the dominant force (e.g. Schumer et al. 2018; Edelman et al. 2019; Martin et al. 2019; Duranton and Pool 2022); conversely, a negative correlation is expected if the foreign ancestry is favored (Pool 2015; Corbett-Detig and Nielsen 2017; Duranton and Pool 2022) or if the effects of deleterious variants are recessive (Kim et al. 2018). Interestingly, we found that the correlation between admixture proportions and recombination rates was in general weak and varied across the Baltic Sea: correlation was slightly positive in Germany, nonexistent in the central Baltic Sea, and weakly negative in the more northern parts. This pattern may have arisen from a historical process that the populations inhabiting the southern Baltic Sea region experienced a more recent influx of WL gene flow, in contrast to the more northern populations which likely retain a more ancestral state of admixture (Feng et al. 2022). This aligns with the observation of a declining LD-based estimate of current *N*_e_ only in the German population (supplementary fig. S1, Supplementary Material online), which is a typical indicator of recent gene flow (Santiago et al. 2020). In addition, our earlier analysis (Feng et al. 2022) revealed a surprising pair of ancestrally related freshwater populations—one in the Baltic Sea side in Latvia and the other in the North Sea side in Tyrifjorden, Norway—with a slightly higher WL ancestry than in the BS7 set. If this level of WL ancestry reflects the ancestral ghost population, the BS7 populations have actually been introgressed from the EL and only the German and Polish populations from the WL. Then, the correlation between the recombination rate and the proportion of the recent introgression, EL in the north and WL in the south, is indeed positive.

On the other hand, such a pattern is also consistent with a model where the selection against introgression varies during the process. At the early stages, the selection against incompatibilities and highly deleterious variation is the dominant force, creating a positive correlation (Duranton and Pool 2022); with increasing distance from the secondary contact zone, the foreign ancestry gets “filtered,” and the force of selection against it diminishes, which in turn weakens the correlation between the recombination rate and the levels of introgression (Duranton and Pool 2022; Groh and Coop 2023). Consistent with this, we found that the purifying selection has very efficiently purged early stop codons and amino-acid-changing variation of WL origin in the southern and central parts of the Baltic Sea, while the selection against the different types of variants tends to become more similar toward the north (Fig. 4). Importantly, at the genome-wide level, we found no evidence of purging of the putatively deleterious moderate- and low-impact variants, and thus, neither drift nor selection has significantly affected the overall proportions of weakly deleterious alleles. The deficit of high-impact coding variants of WL origin in the southern Baltic Sea is surprisingly strong (Fig. 4); as the WL-origin variants appear at a frequency > 0.95 in the North Sea population, they cannot be highly deleterious in that environment. The strong selection against them indicates that they must be maladaptive on the Baltic Sea side and suggests that the environmental differences are the driver of this selection.

The observed enrichment of introgression within promoter regions (Fig. 2a) is contrary to findings in human studies (Petr et al. 2019; Telis et al. 2020) but agrees with those in swordtail fishes (Schumer et al. 2018). In swordtail fishes, recombination is concentrated within promoter and other functional regions (Baker et al. 2017), whereas in humans, the process is driven by specific DNA motifs detected by the chromatin-modifying protein PRDM9 (Myers et al. 2010). The relationship between recombination and genomic features in the 9-spined stickleback is unknown, and recombination cannot be excluded as a factor explaining the high WL ancestry within promoters. On the other hand, introgression is more likely to induce regulatory changes in gene expression than radical alterations in protein-coding genes (Gittelman et al. 2016; Dannemann et al. 2017; Dannemann and Kelso 2017; McCoy et al. 2017; but see Petr et al. (2019) and Telis et al. (2020)). If so, the strong and consistent pattern across all populations could suggest that a selective sweep happened in the first admixture event between the EL and the ancestral Baltic Sea population. The opposite (and consistent) trends for the classes “Promoter” and “CE In Gene” in WL ancestry are striking and may suggest that genetic incompatibilities take place on the level of coding genes (selecting against minor parental ancestry; here WL), while the adaption is driven by gene regulation (favoring the local ancestry; here ancestral Baltic Sea of WL origin).

### Adaptive Introgression

Recent studies suggest that introgression is an important source of genetic variation and allows adaptive evolution to proceed much faster than it would do with de novo mutations (Racimo et al. 2015; Edelman and Mallet 2021). Evidence for AI has been found in a diverse array of taxa, including humans (e.g. Racimo et al. 2015), fish (e.g. Meier et al. 2017; Oziolor et al. 2019), butterflies (Pardo-Diaz et al. 2012; The Heliconius Genome Consortium 2012), and plants (Whitney et al. 2006). Our results add to this evidence by identifying 4 genes with high-frequency WL-origin variants in the EL genetic backgrounds with footprints of selection.

Two of these genes might be associated with reproduction. The zona pellucida glycoprotein 4 (ZP4) gene is known for its importance in sperm–egg interactions during fertilization (Wassarman et al. 2001). As a key component of the fish chorion (egg cell coat), it could have a role in the adaptation to the brackish water conditions of the Baltic Sea (Lønning and Solemdal 1972; Nissling 2002; Jovine et al. 2005). The second gene, dynein axonemal heavy chain 5 (DNAH5), encodes dyneins, essential for flagellar beating and sperm function (Turner 2003). Harmful mutations in the DNAH5 gene have been associated with dysfunction in spermatozoa (Zuccarello et al. 2008). Notably, it is not the first time that DNAH5 has emerged as a candidate gene behind salinity-associated ecological speciation in the Baltic Sea: The footprints of selection in DNAH5 suggest that the gene has contributed to the flounders’ adaptation to low-salinity Baltic Sea conditions and allow sperm activation in lower salinities than in the related saltwater-adapted European flounder (Momigliano et al. 2017; Jokinen et al. 2019).

The relatively small number of positively selected candidates from our study may be an underestimate due to challenges in identifying positively selected introgressed variants and our rather stringent approach to screening for candidates of AI. In contrast to some other studies (e.g. Teng et al. 2017; Walsh et al. 2018; Hu et al. 2019), we first identified regions showing enrichment of introgressed variants and then located the candidate regions with signatures of positive selection on the introgressed variants. This approach might have rendered our ability to identify candidates for AI conservatively, and further work is needed to identify the actual targets of selection and biological functions of the candidate genes and promoters. Nevertheless, it seems reasonable that the introgressed WL-origin variants have played a key role in the adaptation of the dominantly EL-origin sticklebacks—with a recent freshwater history in the northern Fennoscandia (Feng et al. 2022)—to the warmest and most saline part of the southern Baltic Sea.

## Conclusions

To conclude, our study of genetic introgression between 2 divergent stickleback lineages in the Baltic Sea demonstrates that the stabilization of hybrid genomes after admixture is a multistage process where the purifying selection against introgressed deleterious variations has played a central role. The varying genomic landscapes of foreign ancestry are likely the consequence of different types and targets of selection and their interactions, as well as the distribution of functional elements and the variation in recombination. Our work adds a well-worked example to studies showing that introgression can contribute to local adaptation, in spite of the widespread evidence suggesting that selection against introgression is pervasive. Although the observed weak correlation between levels of introgression and recombination rate is in stark contrast to findings in most earlier studies (e.g. Schumer et al. 2018; Edelman et al. 2019; Martin et al. 2019; Stankowski et al. 2019), it highlights the complexity of selection on shaping the genomic landscape of introgression since the occurrence of the admixture event. While more work is needed to distinguish the different forces shaping the ancestry of hybrid genomes, our findings bring new insights into the formation of a heterogeneous landscape of introgression and highlight the importance of considering demographic history, genome structure, parental population differentiation, as well as recombination rate in understanding introgression.

## Materials and Methods

### Ethics Statement

The data used in this study were collected in a previous study (Feng et al. 2022) and in accordance with the national legislation of the countries concerned.

### Data Acquisition

The data were subsetted from the vcf file provided in Feng et al. (2023) using BCFtools v.1.7 (Li et al. 2009). Following Feng et al. (2022), DEN-NOR (from the North Sea) was used as the WL source population in both *f_4_-ratio* and *fd* tests (see below), GBR-GRO (from the UK) as the *WL reference* population for the *WL source* population in the *f_4_-ratio* test (see below), and RUS-LEV (from the White Sea) as the EL source population in both *f_4_-ratio* and *fd* tests, and CAN-TEM (from Quebec, Canada) was selected as the outgroup. As the focal study populations, 9 admixed marine populations from the Baltic Sea identified by Feng et al. (2022) were selected (Fig. 1a and supplementary table S1, Supplementary Material online). In the earlier analyses of this data (Feng et al. 2023), the reads were first mapped to the latest 9-spined stickleback reference genome (Kivikoski et al. 2021) using the Burrows-Wheeler Aligner v.0.7.17 (BWA-MEM algorithm, Li, 2013) and its default parameters. Duplicate reads were marked with SAMtools v.1.7 (Li et al. 2009), and variant calling was performed with the Genome Analysis Toolkit (GATK) v.4.0.1.2 (McKenna et al. 2010) following the GATK Best Practices workflows. Sites located within identified repetitive sequences (Varadharajan et al. 2019) and negative mappability mask regions combining the identified repeats and unmappable regions (Kivikoski et al. 2021) were excluded. Multiallelic variants and sites showing an extremely low (<5×) or high average coverage (>25×), genotype quality score (GQ) < 20, quality score (QUAL) < 30, and missing data >75% were filtered out using VCFtools v.0.1.5 (Danecek et al. 2011). The 2 lineages are known to have distinct sex chromosomes (LG12 for EL and unconfirmed for WL), and data from the known sex chromosomes (LG12, Natri et al. 2019) were removed from further analysis (Feng et al. 2022).

During the finalization of this study, the WL sex determination region was discovered to be located in an 80 kbp inversion in LG3 (Yi et al. 2023). Given its inversion status and the small size (the EL sex chromosome makes up some 50.5% of the 33.6-Mbp-long LG12), we do not believe that its inclusion to our analyses has much influence on the results.

### Quantification of Genomic Introgression

The *f_4_-ratio* test (Reich et al. 2009) was applied to quantify the amount of foreign ancestry in different genomic features. Following Petr et al. (2019) and Feng et al. (2022), the WL ancestry (α_WL_) was estimated as:


(1) $$\begin{array}{r} \alpha_{WL}=\frac{\;f_{4}(\mathrm{GBR}\text{-}\mathrm{GRO},\;\mathrm{CAN}\text{-}\mathrm{TEM};\;\mathrm{Test},\;\mathrm{RUS}\text{-}\mathrm{LEV})}{\;f_{4}(\mathrm{GBR}\text{-}\mathrm{GRO},\;\mathrm{CAN}\text{-}\mathrm{TEM};\;\mathrm{DEN}\text{-}\mathrm{NOR},\;\mathrm{RUS}\text{-}\mathrm{LEV})} \end{array}.$$

*f_4_-ratio* tests were performed using ADMIXTOOLS v.5.1 (qpF4ratio v.320; Patterson et al. 2012). In brief, this setup (Fig. 2b) assumes that the WL population that contributed to the Baltic Sea populations formed a clade with DEN-NOR, rather than with the more ancestral GBR-GRO (Feng et al. 2022), enabling us to directly measure the contribution of the WL source population to the Baltic Sea test populations. For further details on the choice of the optimal WL source, see Feng et al. (2022).

To estimate the admixture proportion among different genomic features, the locations of constrained elements were lifted from the 3-spined stickleback genome annotation (Ensembl release ver. 95; Herrero et al. 2016) using CrossMap v.0.3.3 (Zhao et al. 2014) and a liftover chain created with LAST (Frith et al. 2010) and the Kent utilities (Tyner et al. 2017). The genome annotations from Varadharajan et al. (2019) were lifted to the latest 9-spined stickleback reference genome (Kivikoski et al. 2021) using liftoff (Shumate and Salzberg 2021). The promoter regions were defined as 1 kb stretches upstream of the gene start. A significance test was then applied to assess whether EL and WL ancestry were significantly enriched or depleted in any of the genomic features in comparison with the levels seen within intergenic regions. Following Petr et al. (2019), the alpha value of a given annotation category was resampled 10,000 times from a normal distribution centered on the alpha with a standard deviation equal to the standard error given by ADMIXTOOLS. An empirical *P*-value was then calculated for the estimated alpha for each genomic feature to test the hypothesis that the ancestry proportions for different genome features do not differ from that of the intergenic regions.

### Quantification of WL Introgression (*fd*) and Population Genetic Statistics

The modified *D*-statistic, *fd* (Martin et al. 2015), was used to quantify introgression for the admixed population at finer genomic scales. We used a fixed window size of 100 kb with a 20 kb step size using the scripts from Martin et al. (2015) and estimated *P*-values from the *Z*-transformed *fd* values using the standard normal distribution and corrected for multiple testing with the Benjamini–Hochberg FDR method (Benjamini and Hochberg 1995). Windows with positive *D* and *fd* values with a number of informative sites ≥ 100 and FDR value ≤ 0.05 were retained as outlier loci (see below). Similarly to the *f4-ratio* test, we used the DEN-NOR and RUS-LEV to represent the WL and EL ancestral population and CAN-TEM as the outgroup. The admixture proportions were estimated separately for each Baltic Sea population and for a combined set of 7 northern populations showing similar levels of introgression (i.e. SWE-GOT, FIN-HEL, FIN-TVA, FIN-HAM, FIN-SEI, SWE-BOL, and FIN-KIV, hereafter BS7 when referred collectively).

We examined the covariation of admixture proportions (*fd*) with the population genetic statistics *π* (nucleotide diversity), *d*_xy_ (absolute divergence), and *F*_ST_ (measure of genetic drift). The statistics were computed genome-wide in 10 and 100 kb windows using the scripts from Martin et al. (2015). The mean recombination rate was estimated from the linkage map Varadharajan et al. (2019), initially for 10 kb windows (see Varadharajan et al. (2019) for details) and then binning the rates into 100 kb nonoverlapping windows. The rates were lifted to the latest 9-spined stickleback reference genome (Kivikoski et al. 2021) using custom scripts. The 10 kb population genetic statistics were used in fine-scale analyses of candidate regions for adaptive evolution.

### Footprints of Selection in Baltic Sea Populations

After the quantification of WL introgression, we searched for footprints of selection on introgressed variants using the *U* and *Q95* tests following Racimo et al. (2016) and Jagoda et al. (2018). Both tests are based on the VAF and measure, respectively, the number of SNPs shared with the donor population that appear at a high frequency in the focal population but at a low frequency in the reference population, and the 95% quantile of the frequency of the SNPs that are shared with the donor population and appear at a low frequency in the reference population. The VAF was estimated separately for WL (DEN-NOR), EL (RUS-LEV), and the Baltic Sea populations, and the tests were performed using 100 kb windows with a 20 kb step and discarding positions with more than 25% missing data. We first calculated the *U20*EL, BALTIC, and WL(0.01, 0.2, 1) to count the SNPs that are at <1% frequency in the EL reference population, at ≥20% frequency in the combined Baltic Sea population (BS7), and fixed (100% frequency) in the WL population. We then calculated the *Q95*EL, BALTIC, and WL(0.01, 0.2, 1) to obtain the 95% quantile VAF of these SNPs in the Baltic Sea population. The intersection of the top 1% regions of the *U20* and *Q95* tests and the candidate regions from the *fd* test were then considered as putative AI regions. Within each AI region, *F*_ST_, *d*_xy_, and *π* were calculated for 10 kb sized windows with a 5 kb step, and genotypes and allele frequencies (minor allele frequency [maf] ≥ 0.05) of variants were used to identify candidates for possible adaptive evolution among the lifted reference gene annotations.

### Assessment of Introgressive Genetic Load and Its Purification in the Baltic Sea

Following the concept of *U20* test, we defined the variants in the Baltic Sea populations to be of WL origin if they showed frequency ≤ 0.05 in the EL reference and frequency ≥ 0.95 in the WL reference. To evaluate the efficacy of selection on potentially deleterious foreign variations introduced via genetic introgression, we employed the *r*_xy_ statistics as described by Xue et al. (2015). The *r*_xy_ statistics compared the allele frequencies of certain categories of variants relative with the levels of neutral variants between populations located at varying distances from the entry to the Baltic Sea. An *r*_xy_ value below 1 indicates a deficiency of the focal alleles in the population farther from the WL introgression entry, suggesting the influence of purifying selection. The effects of variants on protein-coding gene sequences were annotated and classified as low impact (synonymous variants), moderate impact (missense variants), and high impact (stop codon gaining variants) using SnpEff v.5.0 (Cingolani et al. 2012). The same number of variants from intergenic regions were randomly selected and served as a proxy for the neutral level of genetic variation. We obtained standard errors and 95% confidence intervals for the *r*_xy_ estimates by jackknifing the values across the 20 individual LGs. For comparison, we also estimated *r*_xy_ for the genome-wide putatively deleterious variants.

## Supplementary Material

Supplementary material online is available at *Molecular Biology and Evolution* online.

## Supplementary Material

msae031_Supplementary_Data

## Data Availability

The whole-genome resequencing data have been published previously in Feng et al. (2022, 2023). All the raw sequence data relevant to this study can be found in European Nucleotide Archive (ENA) (https://www.ebi.ac.uk/ena) through accession code PRJEB39599. Other relevant data can be found in the Zenodo Open Repository: https://zenodo.org/records/10652821.
