# Supplementary material for: Secondary Contact, Introgressive Hybridization, and Genome Stabilization in Sticklebacks
Source: Mol Biol Evol. 2024 Feb 15;41(2):msae031. doi: 10.1093/molbev/msae031 (PMC10903534; doi:10.1093/molbev/msae031)
Supplement: msae031_Supplementary_Data [file msae031_supplementary_data.zip › Supplemental Figures and Tables FINAL.pdf]

Supplemental Figures for manuscript:

# Secondary contact, introgressive hybridization and genome stabilization in sticklebacks

Xueyun Feng[1,2], Juha Merilä[1,3] & Ari Löytynoja[2]

[1]Organismal and Evolutionary Biology Research Programme, Faculty of Biological and Environmental Sciences, 00014 University of Helsinki, Finland.

[2]Institute of Biotechnology, University of Helsinki, 00014 Helsinki, Finland.

[3]Area of Ecology and Biodiversity, The School of Biological Sciences, Kadoorie Biological Sciences Building, The University of Hong Kong, Pokfulam Road, Hong Kong, SAR

Corresponding author (xue-yun.feng@helsinki.fi)

**Supplemental Figures**

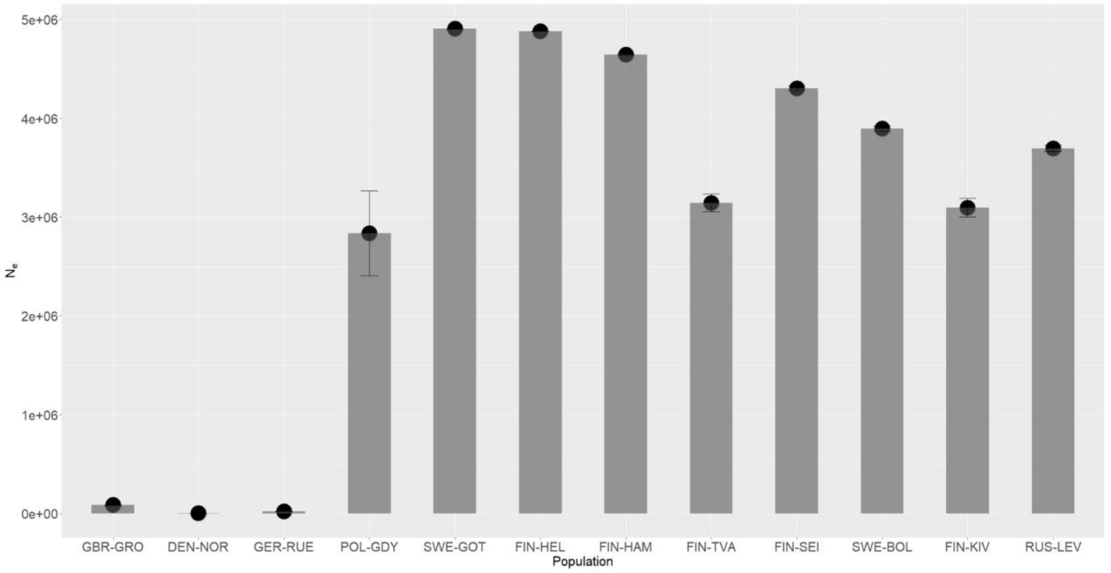


Supplemental Figure S1. The linkage disequilibrium-based estimates of the near-contemporary $N_e$ values and their corresponding confidence intervals for the studied populations, adapted from Feng et al. (2023).

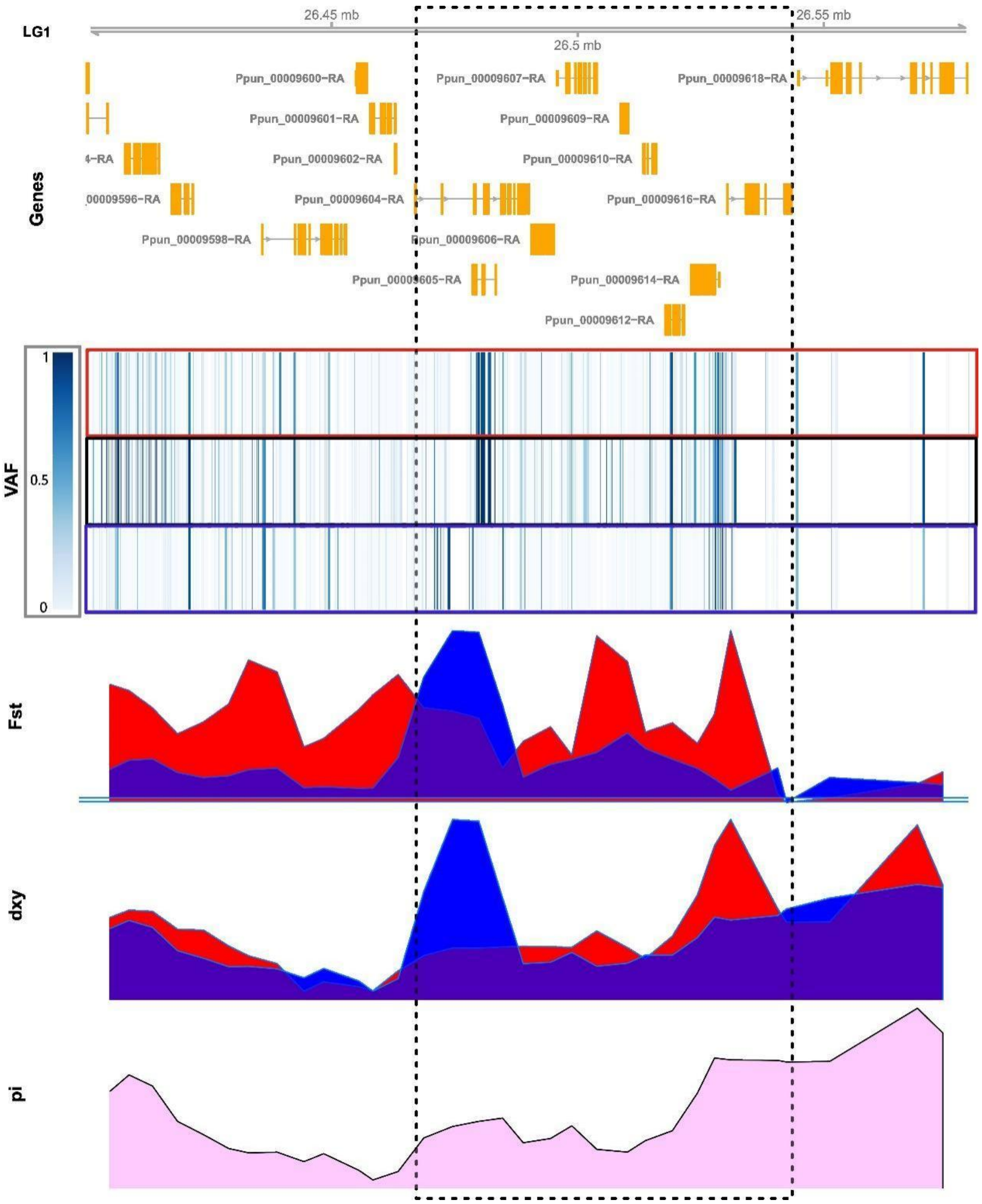
LG1
26.45 mb
26.5 mb
26.55 mb
Genes
Ppun_00009600-RA
Ppun_00009601-RA
Ppun_00009602-RA
Ppun_00009604-RA
Ppun_00009598-RA
Ppun_00009605-RA
Ppun_00009606-RA
Ppun_00009607-RA
Ppun_00009609-RA
Ppun_00009610-RA
Ppun_00009612-RA
Ppun_00009614-RA
Ppun_00009616-RA
Ppun_00009618-RA
VAF
1
0.5
0
Fst
dxy
pi

18

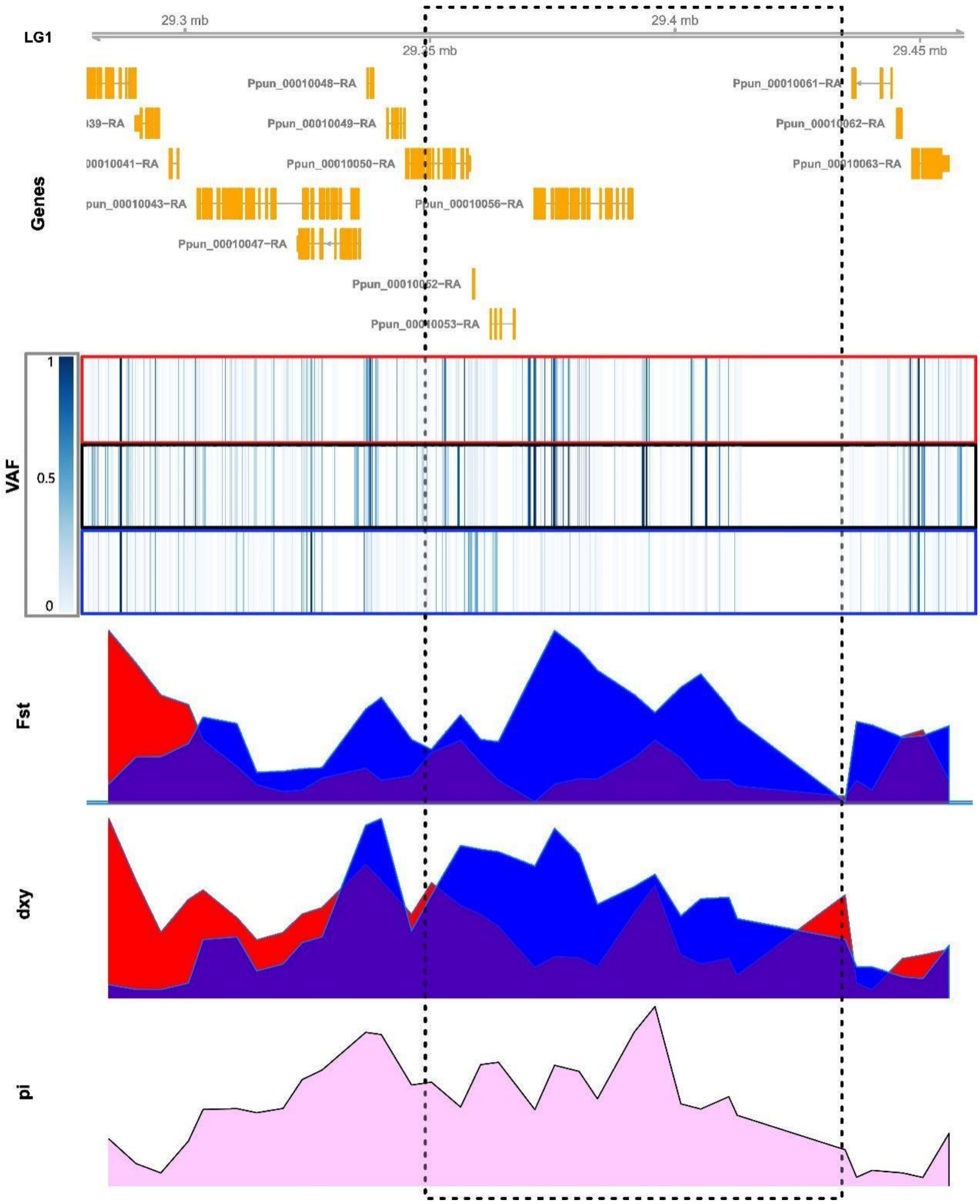

LG1
29.3 mb
29.4 mb
29.35 mb
29.45 mb
Genes
Ppun_00010048−RA
Ppun_00010049−RA
Ppun_00010050−RA
Ppun_00010043−RA
Ppun_00010056−RA
Ppun_00010047−RA
Ppun_00010052−RA
Ppun_00010053−RA
Ppun_00010061−RA
Ppun_00010062−RA
Ppun_00010063−RA
VAF
1
0.5
0
Fst
dxy
pi

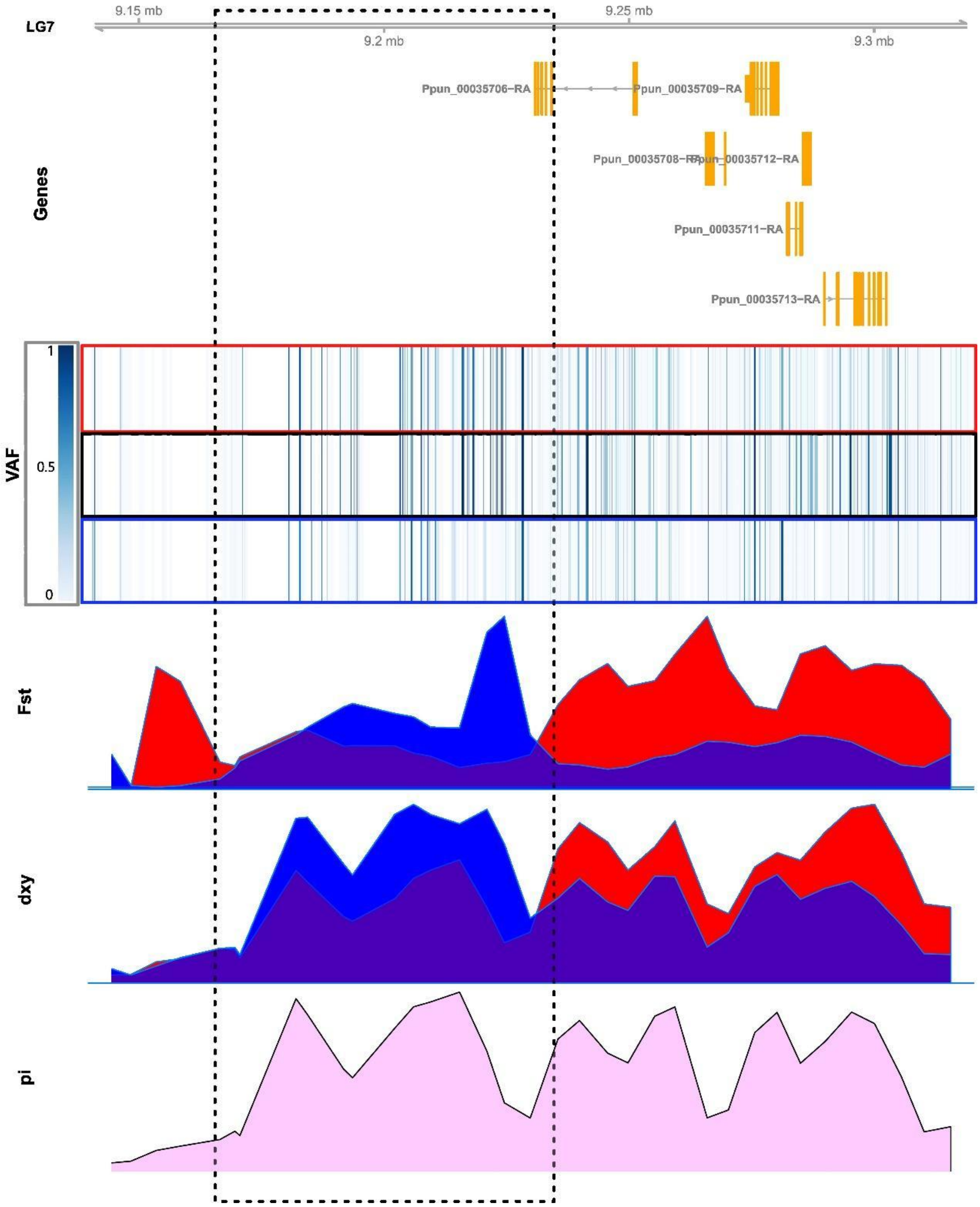

LG7
9.15 mb
9.2 mb
9.25 mb
9.3 mb
Genes
Ppun_00035706-RA
Ppun_00035709-RA
Ppun_00035708-RA
Ppun_00035712-RA
Ppun_00035711-RA
Ppun_00035713-RA
VAF
1
0.5
0
Fst
dxy
pi


20

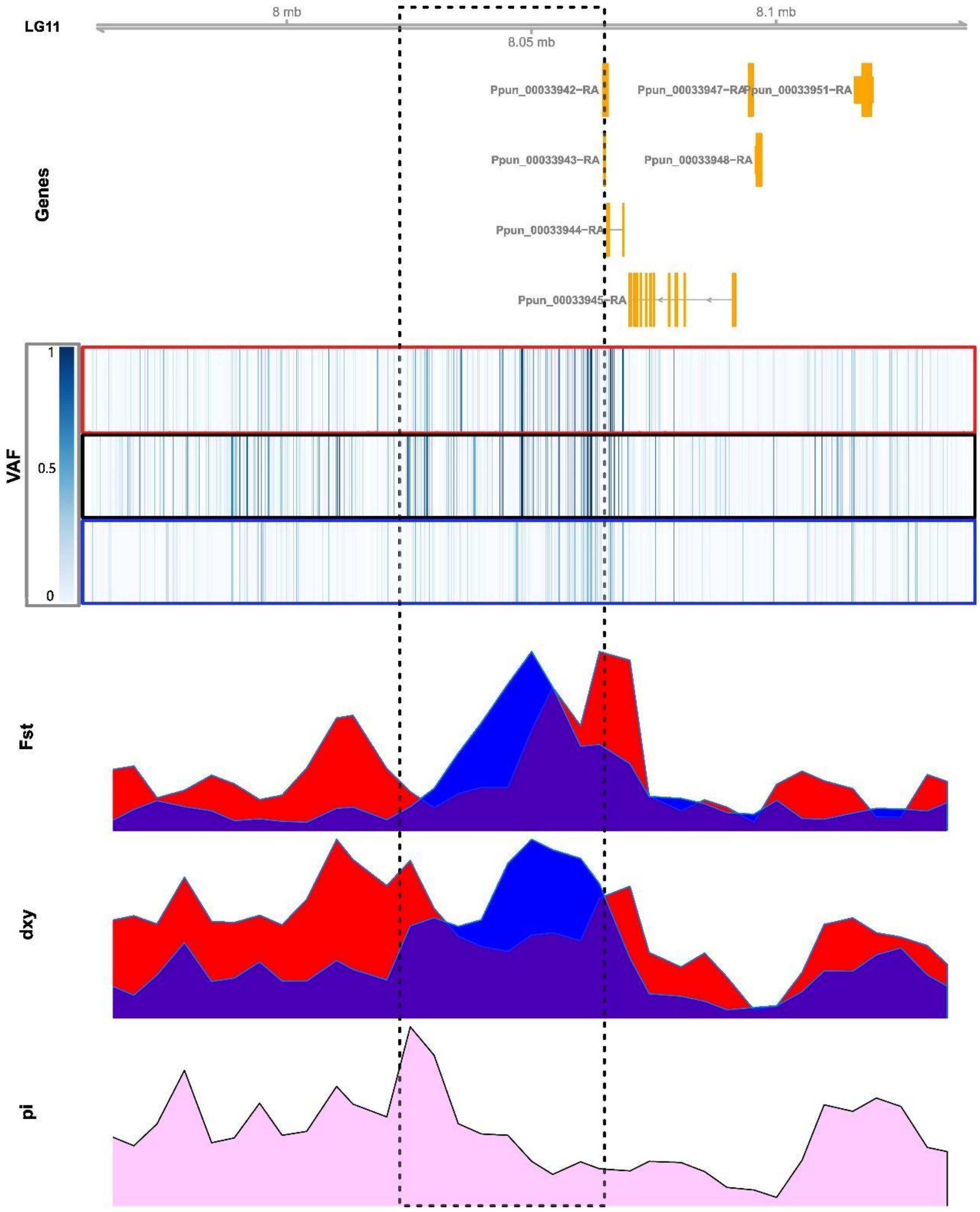


Supplementary Figure S2. Four candidate regions of adaptive introgression identified with *fd*, *U20* and *Q95* analyses (Methods). The panels show the gene annotations (coding sequences in orange), per site variant allele frequencies (VAF; heatmap) for the three sets of population (WL source, BS7, EL source), and $F_{ST}$, $d_{xy}$ and $\pi$ (10kb-windows) in the northern Baltic Sea populations (BS7). The red and blue colors indicate $F_{ST}$ and $d_{xy}$ measured against DEN-NOR (WL source) and RUS-LEV (EL source), respectively.

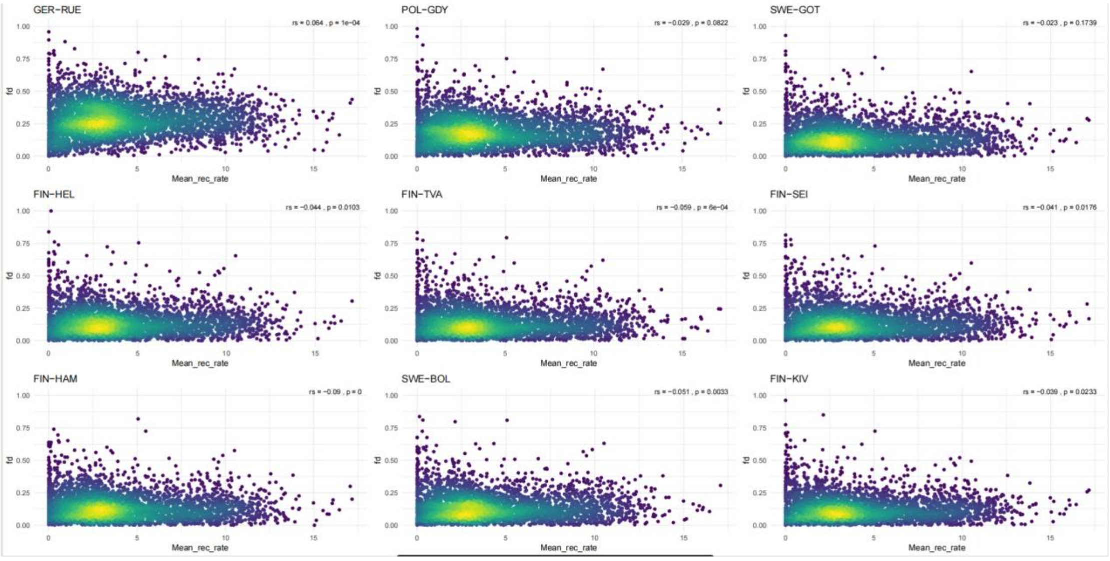


Supplementary Figure S3. Correlations between admixture proportions (fd, 100kb windowed; y-axis) and recombination rates (x-axis). Colors indicate the density of points from low (dark blue) to high (yellow). Each point represents a 100 kb-sized non-overlapping genomic window.

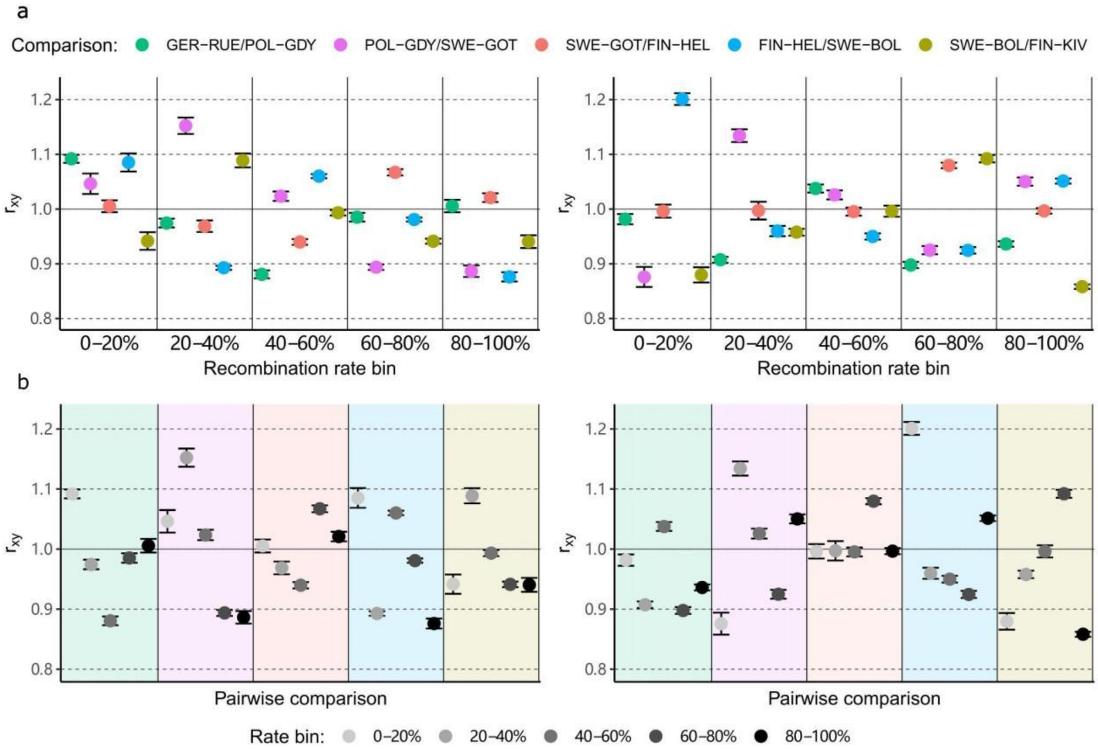


Supplementary Figure S4. The $r_{xy}$ statistics for coding variants (left panels) and promoter region variants (right panels) within the five different recombination rate bins. The panels show (a) $r_{xy}$ for the five pairwise comparisons within each recombination rate bin (Spearman's *rs*=−0.176, *p*=0.220), and (b) $r_{xy}$ for the five recombination rate bins within each pairwise comparison.
