# Supplementary material for: Secondary Contact, Introgressive Hybridization, and Genome Stabilization in Sticklebacks
Source: Mol Biol Evol. 2024 Feb 15;41(2):msae031. doi: 10.1093/molbev/msae031 (PMC10903534; doi:10.1093/molbev/msae031)
Supplement: msae031_Supplementary_Data [file msae031_supplementary_data.zip › Supplemental Tables.pdf]

Tabel S1. Sample information							
Sample information							
Population	Latitude	Longitude	Habitat	Region	Number of individual	Sample year	MT DNA lineage
DEN-NOR	54.98	8.66	Coastal Freshw	North Sea	25	2011	WL
FIN-HAM	60.56	27.19	Marine	Baltic Sea	20	2009	MIX
FIN-HEL	60.2	25.18	Marine	Baltic Sea	22	2007/2008/2010	MIX
FIN-KIV	65	25.47	Marine	Baltic Sea	19	2003	MIX
FIN-SEI	60.23	21.95	Marine	Baltic Sea	24	2010	MIX
FIN-TVA	59.83	23.2	Marine	Baltic Sea	22	2009	MIX
GBR-GRO	57.62	-7.51	Coastal Freshw	British Isles	19	2015	WL
GER-RUE	54.01	13	Marine	Baltic Sea	27	2009	MIX
POL-GDY	54.4	18.53	Marine	Baltic Sea	20	2009	MIX
RUS-LEV	66.3	33.4	Marine	White Sea	30	2007	EL
SWE-BOL	63.66	20.21	Marine	Baltic Sea	21	2007	MIX
SWE-GOT	57.73	18.95	Marine	Baltic Sea	19	2011	MIX
CAN-TEM	47.71	-68.91	Lake	Canadian Inland	16	2010	NA

Table S2. Result of f4-ratio test and enrichment or depletion of ancestry proportion (alpha) in different genomic categories of admixed populations.

A	B	C	X	O	alpha	std	Z	feature	p.alpha_higher	p.alpha_lower
GBR-GRO	DEN-NOR	RUS-LEV	GER-RUE	CAN-TEM	0.339691	0.007386	45.991	CDS	0.928	0.072
GBR-GRO	DEN-NOR	RUS-LEV	GER-RUE	CAN-TEM	0.335367	0.009545	35.134	CE_ALL	0.941	0.058
GBR-GRO	DEN-NOR	RUS-LEV	GER-RUE	CAN-TEM	0.326738	0.011911	27.433	CE_IN_GENE	0.974	0.026
GBR-GRO	DEN-NOR	RUS-LEV	GER-RUE	CAN-TEM	0.353523	0.015661	22.573	CE_NOT_IN_GE	0.424	0.576
GBR-GRO	DEN-NOR	RUS-LEV	GER-RUE	CAN-TEM	0.364377	0.0082	44.439	PROMOTER	0.048	0.952
GBR-GRO	DEN-NOR	RUS-LEV	GER-RUE	CAN-TEM	0.342347	0.00465	73.618	INTRON	0.961	0.039
GBR-GRO	DEN-NOR	RUS-LEV	GER-RUE	CAN-TEM	0.350517	0.004901	71.523	INTERGENIC	1.000	1.000
GBR-GRO	DEN-NOR	RUS-LEV	POL-GDY	CAN-TEM	0.219545	0.006801	32.283	CDS	0.655	0.344
GBR-GRO	DEN-NOR	RUS-LEV	POL-GDY	CAN-TEM	0.207888	0.009144	22.736	CE_ALL	0.942	0.058
GBR-GRO	DEN-NOR	RUS-LEV	POL-GDY	CAN-TEM	0.20437	0.011138	18.349	CE_IN_GENE	0.945	0.055
GBR-GRO	DEN-NOR	RUS-LEV	POL-GDY	CAN-TEM	0.215267	0.014984	14.367	CE_NOT_IN_GE	0.683	0.317
GBR-GRO	DEN-NOR	RUS-LEV	POL-GDY	CAN-TEM	0.238587	0.007851	30.388	PROMOTER	0.018	0.982
GBR-GRO	DEN-NOR	RUS-LEV	POL-GDY	CAN-TEM	0.219753	0.004248	51.727	INTRON	0.730	0.269
GBR-GRO	DEN-NOR	RUS-LEV	POL-GDY	CAN-TEM	0.22231	0.004498	49.425	INTERGENIC	1.000	1.000
GBR-GRO	DEN-NOR	RUS-LEV	SWE-GOT	CAN-TEM	0.142995	0.006206	23.041	CDS	0.093	0.907
GBR-GRO	DEN-NOR	RUS-LEV	SWE-GOT	CAN-TEM	0.120607	0.008843	13.638	CE_ALL	0.946	0.054
GBR-GRO	DEN-NOR	RUS-LEV	SWE-GOT	CAN-TEM	0.11682	0.010518	11.106	CE_IN_GENE	0.956	0.044
GBR-GRO	DEN-NOR	RUS-LEV	SWE-GOT	CAN-TEM	0.128912	0.015354	8.396	CE_NOT_IN_GE	0.652	0.348
GBR-GRO	DEN-NOR	RUS-LEV	SWE-GOT	CAN-TEM	0.160817	0.00739	21.76	PROMOTER	0.000	1.000
GBR-GRO	DEN-NOR	RUS-LEV	SWE-GOT	CAN-TEM	0.137351	0.003807	36.082	INTRON	0.259	0.741
GBR-GRO	DEN-NOR	RUS-LEV	SWE-GOT	CAN-TEM	0.134821	0.00413	32.643	INTERGENIC	1.000	1.000
GBR-GRO	DEN-NOR	RUS-LEV	FIN-HEL	CAN-TEM	0.134032	0.005941	22.56	CDS	0.134	0.866
GBR-GRO	DEN-NOR	RUS-LEV	FIN-HEL	CAN-TEM	0.116254	0.008146	14.272	CE_ALL	0.913	0.086
GBR-GRO	DEN-NOR	RUS-LEV	FIN-HEL	CAN-TEM	0.113883	0.009934	11.464	CE_IN_GENE	0.910	0.090
GBR-GRO	DEN-NOR	RUS-LEV	FIN-HEL	CAN-TEM	0.121779	0.014136	8.615	CE_NOT_IN_GE	0.651	0.349
GBR-GRO	DEN-NOR	RUS-LEV	FIN-HEL	CAN-TEM	0.153405	0.006964	22.028	PROMOTER	0.000	1.000
GBR-GRO	DEN-NOR	RUS-LEV	FIN-HEL	CAN-TEM	0.128519	0.003715	34.591	INTRON	0.376	0.624
GBR-GRO	DEN-NOR	RUS-LEV	FIN-HEL	CAN-TEM	0.127359	0.003888	32.753	INTERGENIC	1.000	1.000
GBR-GRO	DEN-NOR	RUS-LEV	FIN-TVA	CAN-TEM	0.137784	0.00635	21.697	CDS	0.126	0.874
GBR-GRO	DEN-NOR	RUS-LEV	FIN-TVA	CAN-TEM	0.115797	0.008373	13.83	CE_ALL	0.964	0.036
GBR-GRO	DEN-NOR	RUS-LEV	FIN-TVA	CAN-TEM	0.111479	0.010195	10.935	CE_IN_GENE	0.969	0.031
GBR-GRO	DEN-NOR	RUS-LEV	FIN-TVA	CAN-TEM	0.125269	0.014858	8.431	CE_NOT_IN_GE	0.644	0.356

GBR-GRO	DEN-NOR	RUS-LEV	FIN-TVA	CAN-TEM	0.158263	0.007321	21.618	PROMOTER	0.000	1.000
GBR-GRO	DEN-NOR	RUS-LEV	FIN-TVA	CAN-TEM	0.13009	0.003856	33.736	INTRON	0.543	0.457
GBR-GRO	DEN-NOR	RUS-LEV	FIN-TVA	CAN-TEM	0.130549	0.004089	31.926	INTERGENIC	1.000	1.000
GBR-GRO	DEN-NOR	RUS-LEV	FIN-HAM	CAN-TEM	0.129368	0.005768	22.43	CDS	0.096	0.903
GBR-GRO	DEN-NOR	RUS-LEV	FIN-HAM	CAN-TEM	0.106083	0.00811	13.081	CE_ALL	0.974	0.026
GBR-GRO	DEN-NOR	RUS-LEV	FIN-HAM	CAN-TEM	0.103366	0.009464	10.922	CE_IN_GENE	0.974	0.026
GBR-GRO	DEN-NOR	RUS-LEV	FIN-HAM	CAN-TEM	0.112034	0.013857	8.085	CE_NOT_IN_GE	0.764	0.236
GBR-GRO	DEN-NOR	RUS-LEV	FIN-HAM	CAN-TEM	0.144532	0.006875	21.021	PROMOTER	0.001	0.999
GBR-GRO	DEN-NOR	RUS-LEV	FIN-HAM	CAN-TEM	0.121556	0.003645	33.349	INTRON	0.522	0.478
GBR-GRO	DEN-NOR	RUS-LEV	FIN-HAM	CAN-TEM	0.12181	0.003892	31.297	INTERGENIC	1.000	1.000
GBR-GRO	DEN-NOR	RUS-LEV	FIN-SEI	CAN-TEM	0.14317	0.006424	22.285	CDS	0.083	0.917
GBR-GRO	DEN-NOR	RUS-LEV	FIN-SEI	CAN-TEM	0.12106	0.008504	14.236	CE_ALL	0.942	0.058
GBR-GRO	DEN-NOR	RUS-LEV	FIN-SEI	CAN-TEM	0.115224	0.010437	11.04	CE_IN_GENE	0.965	0.035
GBR-GRO	DEN-NOR	RUS-LEV	FIN-SEI	CAN-TEM	0.133605	0.015114	8.84	CE_NOT_IN_GE	0.518	0.482
GBR-GRO	DEN-NOR	RUS-LEV	FIN-SEI	CAN-TEM	0.158836	0.007548	21.043	PROMOTER	0.001	0.999
GBR-GRO	DEN-NOR	RUS-LEV	FIN-SEI	CAN-TEM	0.135548	0.003989	33.98	INTRON	0.380	0.620
GBR-GRO	DEN-NOR	RUS-LEV	FIN-SEI	CAN-TEM	0.134317	0.004485	29.95	INTERGENIC	1.000	1.000
GBR-GRO	DEN-NOR	RUS-LEV	SWE-BOL	CAN-TEM	0.125015	0.005877	21.273	CDS	0.322	0.678
GBR-GRO	DEN-NOR	RUS-LEV	SWE-BOL	CAN-TEM	0.112854	0.008354	13.51	CE_ALL	0.876	0.124
GBR-GRO	DEN-NOR	RUS-LEV	SWE-BOL	CAN-TEM	0.109647	0.009893	11.083	CE_IN_GENE	0.902	0.098
GBR-GRO	DEN-NOR	RUS-LEV	SWE-BOL	CAN-TEM	0.120061	0.014098	8.516	CE_NOT_IN_GE	0.562	0.438
GBR-GRO	DEN-NOR	RUS-LEV	SWE-BOL	CAN-TEM	0.141281	0.006988	20.217	PROMOTER	0.003	0.997
GBR-GRO	DEN-NOR	RUS-LEV	SWE-BOL	CAN-TEM	0.121739	0.003533	34.459	INTRON	0.558	0.442
GBR-GRO	DEN-NOR	RUS-LEV	SWE-BOL	CAN-TEM	0.122237	0.003974	30.757	INTERGENIC	1.000	1.000
GBR-GRO	DEN-NOR	RUS-LEV	FIN-KIV	CAN-TEM	0.116175	0.005681	20.45	CDS	0.274	0.726
GBR-GRO	DEN-NOR	RUS-LEV	FIN-KIV	CAN-TEM	0.101178	0.007673	13.186	CE_ALL	0.933	0.067
GBR-GRO	DEN-NOR	RUS-LEV	FIN-KIV	CAN-TEM	0.097253	0.009409	10.337	CE_IN_GENE	0.948	0.052
GBR-GRO	DEN-NOR	RUS-LEV	FIN-KIV	CAN-TEM	0.109708	0.013014	8.43	CE_NOT_IN_GE	0.591	0.409
GBR-GRO	DEN-NOR	RUS-LEV	FIN-KIV	CAN-TEM	0.132934	0.006719	19.786	PROMOTER	0.002	0.998
GBR-GRO	DEN-NOR	RUS-LEV	FIN-KIV	CAN-TEM	0.110996	0.003399	32.657	INTRON	0.696	0.304
GBR-GRO	DEN-NOR	RUS-LEV	FIN-KIV	CAN-TEM	0.112718	0.00382	29.505	INTERGENIC	1.000	1.000

Table S3. Detailed information for each collapsed candidate region identified by fd analysis.

Chr	Start	End
LG1	6840001	6960000
LG1	26420001	26560000
LG1	29300001	29500000
LG3	14200001	14300000
LG3	15680001	15800000
LG4	660001	760000
LG4	24080001	24260000
LG4	26060001	26160000
LG5	9400001	9540000
LG6	560001	660000
LG6	11820001	11920000
LG6	17940001	18220000
LG7	2100001	2340000
LG7	9100001	9280000
LG7	17360001	17480000
LG8	10800001	10960000
LG8	20280001	20440000
LG9	8880001	9080000
LG9	11800001	12000000
LG9	19560001	19660000
LG10	1220001	1320000
LG10	4300001	4520000
LG10	11180001	11740000
LG11	8020001	8140000
LG11	12580001	13100000
LG11	17740001	17840000
LG14	2460001	2620000
LG14	14560001	14660000

LG14	16120001	16380000			
LG16	5160001	5260000			
LG17	6840001	6940000			
LG18	540001	660000			
LG18	2960001	3120000			
LG19	20320001	20440000			
LG20	600001	720000			
LG20	2080001	2180000			
LG20	10500001	10940000			
LG20	15920001	16020000			
LG20	20240001	20340000			
LG20	21060001	21220000			
LG20	21280001	21380000			
LG21	9120001	9240000			
LG21	9940001	10180000			
LG21	10420001	10540000			
LG21	10900001	11000000			

Table S4. Collapsed candidate regions identified by U and Q95 test;and genes in each region.

Chr	Start	End	Overlapped with fd	Genes
LG1	20100001	20280000	no	clptm1;RELB;mrpl28;Slc30a1;ppp1r37;H2A;bmp4;GEMIN7;calm1;Ptgir;Ppm1a;Rtn2;Nlrx1
LG1	26400001	26580000	yes	SERPINH1;GDPD5;HSPA13;SAMSN1;ZP4;P2RX5;Dhx40;AKT2;CNTD2;CIPC;SLC6A13;Vwa7;VWA5A
LG1	29280001	29460000	yes	NLK;smco4;NF1;SWP2;COX7A2;Ribonuclease Oy;supt5h;TRIAP1;IL15;Plekhg3;P2RY14;SHKBP1
LG3	3860001	4060000	no	ADGRL2;PRKACB;Gpaa1;rbck1;GPT;DHX30;Cela3b;WRNIP1;BEST4;myadm;Nfatc4;LTB4R;SDR39U1;METTL17;Parp2;Myl3;
LG7	9140001	9320000	yes	UBL3;Hmgb1;uspl1;ALOX5AP;Medag;B3GLCT
LG9	2680001	2860000	no	RASD2;PRPSAP1;PIK3R6;PIK3R5;NTN1;STX8
LG9	4840001	5020000	no	PCDH7; CRMP1; Wfs1;PPP2R2C; PDE5A;ANK2;camk2d2
LG11	7960001	8140000	yes	SSTR2;usp22; med9;RASD1;sox8
LG14	10500001	10620000	no	ASTN2;TRIM32
LG14	14280001	14460000	no	Ankra2; SREK1; Lifr; Mast4; PIK3R1; TNC;TRAF2;GRAMD3;znf703
LG20	20220001	20340000	yes	trio;DNAH5;ADCY2;PAPD7

Table S5. Correlation between measure of genetic drift (FST), absolute genetic distance (dxy) and recombination rate			
Population	Spearman's rs	p-value	Comparison between recombination rate and
GER-RUE	0.19	1.36E-32	FST to DEN-NOR
GER-RUE	0.237	2.86E-50	FST to DEN-NOR
GER-RUE	0.532	4.09E-280	dxy to RUS-LEV
GER-RUE	0.589	0	dxy to DEN-NOR
POL-GDY	0.212	2.97E-40	FST to DEN-NOR
POL-GDY	0.281	1.34E-70	FST to DEN-NOR
POL-GDY	0.486	6.91E-228	dxy to RUS-LEV
POL-GDY	0.607	0	dxy to DEN-NOR
FIN-HEL	0.187	1.72E-31	FST to DEN-NOR
FIN-HEL	0.286	2.09E-73	FST to DEN-NOR
FIN-HEL	0.472	1.92E-213	dxy to RUS-LEV
FIN-HEL	0.615	0	dxy to DEN-NOR
FIN-TVA	0.193	8.35E-34	FST to DEN-NOR
FIN-TVA	0.299	3.51E-80	FST to DEN-NOR
FIN-TVA	0.466	3.58E-207	dxy to RUS-LEV
FIN-TVA	0.613	0	dxy to DEN-NOR
FIN-HAM	0.179	3.59E-29	FST to DEN-NOR
FIN-HAM	0.31	1.18E-86	FST to DEN-NOR
FIN-HAM	0.45	4.71E-191	dxy to RUS-LEV
FIN-HAM	0.61	0	dxy to DEN-NOR
FIN-SEI	0.217	3.24E-42	FST to DEN-NOR
FIN-SEI	0.293	3.69E-77	FST to DEN-NOR
FIN-SEI	0.467	1.52E-207	dxy to RUS-LEV
FIN-SEI	0.611	0	dxy to DEN-NOR
SWE-BOL	0.175	8.09E-28	FST to DEN-NOR
SWE-BOL	0.295	3.06E-78	FST to DEN-NOR
SWE-BOL	0.473	6.21E-214	dxy to RUS-LEV
SWE-BOL	0.614	0	dxy to DEN-NOR

SWE-GOT	0.216	6.52E-42	FST to DEN-NOR			
SWE-GOT	0.285	8.55E-73	FST to DEN-NOR			
SWE-GOT	0.478	3.75E-219	dxy to RUS-LEV			
SWE-GOT	0.609	0	dxy to DEN-NOR			
FIN-KIV	0.141	1.63E-18	FST to DEN-NOR			
FIN-KIV	0.289	3.40E-75	FST to DEN-NOR			
FIN-KIV	0.464	2.12E-205	dxy to RUS-LEV			
FIN-KIV	0.612	0	dxy to DEN-NOR			

Table S6. Comparison of rxy estimated for coding variants with different origins and impact levels								
Population	rxy	SE	Z	p-value	95% CI	Origin	feature	nSNP
GER-RUE v.s. POL-GDY	0.364	0.006	-114.844	0.000	[0.353-0.375]	WL-origin Coding Variants	High	5
POL-GDY v.s. SWE-GOT	0.536	0.011	-41.355	0.000	[0.514-0.558]	WL-origin Coding Variants	High	4
SWE-GOT v.s. FIN-HEL	1.098	0.026	3.712	0.000	[1.046-1.15]	WL-origin Coding Variants	High	5
FIN-HEL v.s. SWE-BOL	0.728	0.006	-47.719	0.000	[0.717-0.739]	WL-origin Coding Variants	High	5
SWE-BOL v.s. FIN-KIV	0.971	0.016	-1.790	0.073	[0.94-1.003]	WL-origin Coding Variants	High	3
GER-RUE v.s. POL-GDY	0.640	0.001	-269.513	0.000	[0.638-0.643]	WL-origin Coding Variants	Moderate	828
POL-GDY v.s. SWE-GOT	0.710	0.002	-188.774	0.000	[0.707-0.713]	WL-origin Coding Variants	Moderate	729
SWE-GOT v.s. FIN-HEL	0.891	0.001	-73.055	0.000	[0.888-0.894]	WL-origin Coding Variants	Moderate	721
FIN-HEL v.s. SWE-BOL	0.936	0.002	-32.995	0.000	[0.932-0.94]	WL-origin Coding Variants	Moderate	687
SWE-BOL v.s. FIN-KIV	0.887	0.001	-81.099	0.000	[0.884-0.89]	WL-origin Coding Variants	Moderate	688
GER-RUE v.s. POL-GDY	0.649	0.001	-423.799	0.000	[0.647-0.65]	WL-origin Coding Variants	Low	1,220
POL-GDY v.s. SWE-GOT	0.706	0.001	-243.989	0.000	[0.704-0.708]	WL-origin Coding Variants	Low	1,089
SWE-GOT v.s. FIN-HEL	0.921	0.001	-67.813	0.000	[0.918-0.923]	WL-origin Coding Variants	Low	1,079
FIN-HEL v.s. SWE-BOL	0.894	0.001	-96.398	0.000	[0.892-0.896]	WL-origin Coding Variants	Low	1,055
SWE-BOL v.s. FIN-KIV	0.948	0.002	-33.024	0.000	[0.945-0.951]	WL-origin Coding Variants	Low	1,032
GER-RUE v.s. POL-GDY	0.651	0.001	-427.424	0.000	[0.649-0.652]	WL-origin Coding Variants	Modifier	5,053
POL-GDY v.s. SWE-GOT	0.708	0.001	-204.762	0.000	[0.705-0.711]	WL-origin Coding Variants	Modifier	4,493
SWE-GOT v.s. FIN-HEL	0.897	0.001	-120.174	0.000	[0.895-0.899]	WL-origin Coding Variants	Modifier	4,493
FIN-HEL v.s. SWE-BOL	0.908	0.001	-83.218	0.000	[0.906-0.91]	WL-origin Coding Variants	Modifier	4,302
SWE-BOL v.s. FIN-KIV	0.923	0.001	-63.718	0.000	[0.921-0.926]	WL-origin Coding Variants	Modifier	4,318
GER-RUE v.s. POL-GDY	0.965	0.003	-13.969	0.000	[0.96-0.97]	Genome-wide Coding Variants	High	367
POL-GDY v.s. SWE-GOT	0.913	0.003	-33.804	0.000	[0.908-0.918]	Genome-wide Coding Variants	High	304
SWE-GOT v.s. FIN-HEL	1.068	0.003	26.474	0.000	[1.063-1.073]	Genome-wide Coding Variants	High	342
FIN-HEL v.s. SWE-BOL	0.996	0.003	-1.189	0.235	[0.99-1.002]	Genome-wide Coding Variants	High	353
SWE-BOL v.s. FIN-KIV	0.978	0.003	-8.584	0.000	[0.973-0.983]	Genome-wide Coding Variants	High	334
GER-RUE v.s. POL-GDY	1.023	0.001	45.466	0.000	[1.022-1.024]	Genome-wide Coding Variants	Moderate	53,687
POL-GDY v.s. SWE-GOT	1.008	0.000	16.547	0.000	[1.007-1.009]	Genome-wide Coding Variants	Moderate	45,821
SWE-GOT v.s. FIN-HEL	0.997	0.000	-13.147	0.000	[0.996-0.997]	Genome-wide Coding Variants	Moderate	48,260
FIN-HEL v.s. SWE-BOL	1.020	0.000	52.237	0.000	[1.02-1.021]	Genome-wide Coding Variants	Moderate	47,125
SWE-BOL v.s. FIN-KIV	0.982	0.000	-56.125	0.000	[0.982-0.983]	Genome-wide Coding Variants	Moderate	46,350
GER-RUE v.s. POL-GDY	1.029	0.000	91.002	0.000	[1.028-1.03]	Genome-wide Coding Variants	Low	74,637

POL-GDY v.s. SWE-GOT	1.016	0.001	27.520	0.000	[1.015-1.017]	Genome-wide Coding Variants	Low	64,259
SWE-GOT v.s. FIN-HEL	0.992	0.000	-41.771	0.000	[0.992-0.993]	Genome-wide Coding Variants	Low	67,395
FIN-HEL v.s. SWE-BOL	1.004	0.000	34.775	0.000	[1.003-1.004]	Genome-wide Coding Variants	Low	64,415
SWE-BOL v.s. FIN-KIV	1.002	0.000	7.147	0.000	[1.001-1.002]	Genome-wide Coding Variants	Low	64,825
GER-RUE v.s. POL-GDY	1.010	0.000	34.165	0.000	[1.009-1.01]	Genome-wide Coding Variants	Modifier	344,690
POL-GDY v.s. SWE-GOT	1.006	0.001	12.161	0.000	[1.005-1.007]	Genome-wide Coding Variants	Modifier	295,779
SWE-GOT v.s. FIN-HEL	0.997	0.000	-14.209	0.000	[0.997-0.997]	Genome-wide Coding Variants	Modifier	306,253
FIN-HEL v.s. SWE-BOL	1.000	0.000	0.982	0.326	[1-1.001]	Genome-wide Coding Variants	Modifier	295,652
SWE-BOL v.s. FIN-KIV	0.998	0.000	-9.430	0.000	[0.997-0.998]	Genome-wide Coding Variants	Modifier	297,184
